# Determination of Cooperativity Length in a Glass-Forming Polymer

**DOI:** 10.1021/acsphyschemau.2c00057

**Published:** 2023-01-04

**Authors:** Yeong Zen Chua, Reiner Zorn, Jürn W. P. Schmelzer, Christoph Schick, Olaf Holderer, Michaela Zamponi

**Affiliations:** † Institute of Physics, University of Rostock, Albert-Einstein-Str. 23-24, 18051Rostock, Germany; ‡ Competence Centre CALOR, Faculty of Interdisciplinary Research, University of Rostock, Albert-Einstein-Str. 25, 18051Rostock, Germany; § Forschungszentrum Jülich GmbH, Jülich Centre for Neutron Science (JCNS-1) and Institute for Biological Information Processing (IBI-8), 52425Jülich, Germany; ∥ Forschungszentrum Jülich GmbH, Jülich Centre for Neutron Science at MLZ, Garching85748, Germany

**Keywords:** glass transition, cooperativity length, thermodynamics, temperature fluctuations, quasi-elastic neutron scattering, AC calorimetry

## Abstract

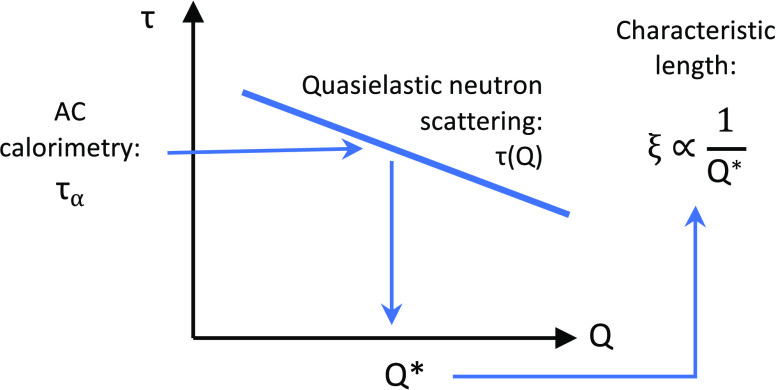

To describe the properties of glass-forming liquids, the concepts of a cooperativity length or the size of cooperatively rearranging regions are widely employed. Their knowledge is of outstanding importance for the understanding of both thermodynamic and kinetic properties of the systems under consideration and the mechanisms of crystallization processes. By this reason, methods of experimental determination of this quantity are of outstanding importance. Proceeding in this direction, we determine the so-called cooperativity number and, based on it, the cooperativity length by experimental measurements utilizing AC calorimetry and quasi-elastic neutron scattering (QENS) at comparable times. The results obtained are different in dependence on whether temperature fluctuations in the considered nanoscale subsystems are either accounted for or neglected in the theoretical treatment. It is still an open question, which of these mutually exclusive approaches is the correct one. As shown in the present paper on the example of poly(ethyl methacrylate) (PEMA), the cooperative length of about 1 nm at 400 K and a characteristic time of ca. 2 μs determined from QENS coincide most consistently with the cooperativity length determined from AC calorimetry measurements if the effect of temperature fluctuations is incorporated in the description. This conclusion indicates that—accounting for temperature fluctuations—the characteristic length can be derived by thermodynamic considerations from the specific parameters of the liquid at the glass transition and that temperature does fluctuate in small subsystems.

## I Introduction

Analysis of the properties of glass-forming liquids and, in particular, their specific features exhibited in the course of the glass transition is a topic of intensive current research. In their theoretical interpretation, the concepts of a cooperativity length or of the size of cooperatively rearranging regions are widely employed. Their knowledge is of outstanding importance for the understanding of both thermodynamic and kinetic properties of the systems under consideration as well as of the mechanisms of crystallization processes. For this reason, the determination of these parameters as a function of temperature or pressure is of outstanding importance in the application of these concepts.

In the present paper, we determine the cooperativity length based on experimental measurements utilizing both AC calorimetry and quasi-elastic neutron scattering (QENS). As the first step, hereby the so-called cooperativity number is determined based on experimental data. However, here a nontrivial problem arises.

The results obtained are different in dependence on whether temperature fluctuations in the considered nanoscale subsystems are either accounted for or neglected. The analysis of this topic, i.e., which of these mutually exclusive approaches is the correct one, has led in the past to considerable theoretical controversies discussed in detail in refs (1) and (2). For example, as noted by Landau and Lifshitz: “Temperature is as entropy obviously a quantity of purely statistical character, which has a definite meaning only for macroscopic systems” (ref (3), Chap. 2, paragraph 9). On the other hand, in treating thermal fluctuations, Landau and Lifshitz consider a small closed subsystem in an extended thermostat (cf. also ref (2)), so the question is obviously how small subsystems can be to be allowed to be treated in such terms.

The opposite point of view was expressed by Kittel, strictly denying the existence of temperature fluctuations. He wrote “the energy of the system may fluctuate but the temperature does not” (Chap. 6, comment to Exercise 6.3 in ref (4)). He retained his point of view also later denoting temperature fluctuations as an oxymoron, i.e., a combination of contradictory words.^5^ This point of view was heavily opposed by Mandelbrot, considering temperature fluctuations as a “well-defined and unavoidable notion”.^6^ Consequently, our analysis also gives an answer to the widely discussed problem of whether temperature fluctuations have to be generally accounted for in thermodynamics or not.

As shown in the present paper, the cooperative length determined from QENS coincides most consistently with the cooperativity length determined from AC calorimetry measurements if the effect of temperature fluctuations is incorporated in the description. This conclusion indicates that—accounting for temperature fluctuations—the characteristic length can be derived by thermodynamic considerations from the specific parameters of the liquid at the glass transition and that temperature does fluctuate in small systems. The latter result may have consequences in the field of Stochastic Thermodynamics when it comes to the treatment of mesoscopic systems.^7^

The paper is structured as follows: First, the strategy of comparing length scales derived from neutron scattering and calorimetry is explained. In the experimental part, the chosen sample and the experimental techniques are briefly described. Then, the experimental results of AC calorimetry and neutron scattering are presented. Finally, the results are combined and discussed in the context of the relevance of temperature fluctuations for nanosized systems. In conclusion, within the still existing limitations of the combination of both methods, this study supports the existence and importance of temperature fluctuations for cooperatively rearranging regions near the glass-transition temperature.

## II Experimental Strategy

In this study, we basically follow the procedure developed and used in ref (1): First, we calculate the cooperativity number *N*_α_ from the measurement of thermodynamic quantities with and without taking temperature fluctuations into account. These numbers are then converted into a characteristic volume via the molecular volume assuming a reasonable shape of the cooperatively rearranging regions (CRR). Second, we determine a length scale from a quasi-elastic neutron scattering (QENS) experiment. The characteristic time from QENS depends on the observed length scale, which is determined by the scattering vector *Q*. By this relation, the time scale of the dynamic glass transition can be associated with a length. For this time scale, the one from AC calorimetry was used. In this paper, we will only briefly recapitulate the most important relations and explain the particularities of the study performed here.

The cooperativity number *N*_α_ neglecting temperature fluctuations is given by^8−11^
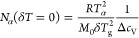
1where Δ*c*_*V*_ = *c*_V_^liquid^ – c_V_^glass^ is the jump of the specific heat capacity at the glass transition and δ*T*_g_ is the width of the distribution of glass-transition temperatures. The corresponding formula accounting for temperature fluctuations is^12,13^
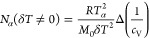
2where Δ(1/*c*_V_) = 1/*c*_V_^glass^ – 1/c_V_^liquid^ is the jump of the *reciprocal* specific heat capacity at the glass transition and δ*T* is the width of the *fluctuation of temperature*. *T*_α_ in both cases is the dynamic glass-transition temperature at a certain frequency. *R* is the universal gas constant, and *M*_0_ is the molecular mass for a low-molecular liquid or the monomer mass for a polymer.

To convert this number into a characteristic length, as in ref (1) we assume a spherical shape of the CRR leading to
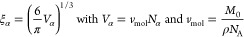
3where *N*_A_ is the Avogadro constant and ρ is the mass density. As in eqs 1 and 2, *M*_0_ is taken as the monomer mass for the polymeric material used here. This assumption may seem debatable because, in a strict chemical sense, the whole chain is the molecule, and for some polymers, the division into monomers is ambiguous. However, this question is irrelevant here since *M*_0_ cancels out in the calculation of ξ_α_.

Because δ*T*_g_ in 1 and δ*T* in 2 are experimentally determined in the same way as the width of the step in the specific heat curve, they both have the same value from experiment. Therefore, in an experimental determination, the two characteristic lengths only differ by a factor weakly depending on temperature
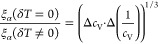
4For the experiment performed here, we were looking for a material with a comparatively large ratio of these length scales because that allows a better resolution of the problem which of the two hypotheses discussed is the correct one. Since also other restrictions (absence of secondary relaxations, suitable value of the glass transition temperature, *T*_g_) had to be taken into account, poly(ethyl methacrylate) (PEMA) emerged as a good candidate for this study. It offers a ratio of 3.5···4.5 in eq 4 instead of ≈ 2 for the earlier studied propylene glycol.^1^

For the derivation of the length scale from QENS, we use the same prescription as in ref (1). By Q* we denoted the scattering vector where the relaxation time from QENS coincides with that of AC calorimetry. Then, the characteristic length is calculated as
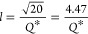
5The argument for this conversion is based on the assumption of a spherical shape of the CRR. It has to be noted that this argument is rather “intuitive” and different values of the numerator were suggested in the literature.^14,15^ The effect of the assumed shape itself is relatively small since it enters eqs 5 and 3 simultaneously, leading to a partial cancellation (see 5). Finally, for the conversion of the frequency value of the maximum of the peak in the imaginary part of the specific heat capacity, ω_max_, into a time scale, we use the maximum of the susceptibility constructed from the relaxation function fitted to the NSE data,^1^ leading to
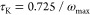
6The slightly different denominator reflects the fact that the stretching exponent of the Kohlrausch function is different here.

## III Experimental Section

### A Sample

PEMA (IUPAC name: poly(ethyl methacrylate) (*M*_0_ = 114.2 g/mol), CAS number: 9003-42-3) was purchased from PolyScience (Hirschberg an der Bergstrasse, Germany). The sample with molecular mass *M*_W_ = 154 kg/mol and *M*_w_/*M*_n_ = 1,9 was used without further purification. PEMA does not crystallize; consequently, no melting peak has been observed upon heating.

**Figure 1:**
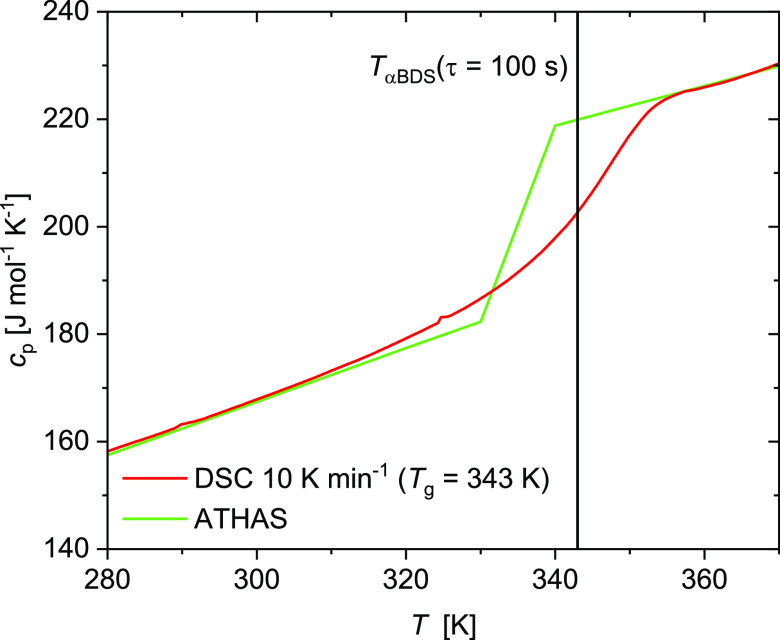
Differential scanning calorimetry (DSC) curve of PEMA at the heating rate 10 K min^–1^. The green line shows the reference data from the ATHAS data bank.^16,17^ The measured values reasonably agree with the reference data; see https://materials.springer.com/polymerthermodynamics/docs/athas_0005.

The glass-transition temperature from DSC at a heating rate of 10 K min^–1^ equals 343 K; see Figure 1. The value is in agreement with *T*_α_ = 343 K at τ_α_ ≈ 100 s, available from dielectric relaxation data collected in a wide frequency range.^18,19^

The specific heat capacities of the glass, the liquid state, and their difference, Δ*c*_*p*_(*T*), change with temperature. For each temperature, the corresponding *c*_*p*_^glass^ and *c*_*p*_^liquid^ were taken from the curves to determine Δ*c*_*p*_ and Δ(1/*c*_*p*_) = 1/*c*_*p*_^glass^ – 1/*c*_*p*_^liquid^ for calculating the cooperativity numbers, as mentioned above. However, for estimating the cooperativity number by eq 1 or 2, the specific heat capacity at constant volume (*c*_V_) is required. For polymers, *c_p_* is only a few percent larger than *c*_v_.^20^ Consequently, the conversion of *c_p_* to *c*_V_ does not significantly affect the difference between the estimates of the cooperative number by eqs 1 and 2.

### B AC Calorimetry

Several experimental techniques allow measuring the complex heat capacity in a broad frequency range. First studies on the relaxation process near the glass transition (α-relaxation) employed the so-called 3ω-technique to obtain the complex effusivity.^21,22^ The accessible frequency range is between 2·10^–3^ and 4·10^3^ Hz.^23^ Combinations of heat capacity and thermal conductivity are also available from other high-frequency methods like photopyroelectric,^24^ thermal lens,^25^ or impulsive stimulated thermal scattering.^26^ Frequencies up to 10^8^ Hz became accessible this way.

The so far mentioned methods provide complex heat capacity and thermal conductivity combinations like complex thermal effusivity. Complex heat capacity curves are preferable for calculating the cooperativity length from calorimetry. A frequency-independent thermal conductivity is commonly assumed when extracting complex heat capacity from effusivity curves. Furthermore, thermodynamics may be coupled with mechanical properties in the corresponding experiments.^27^ This circle of problems was considered using spherical geometries but resulted in a very limited frequency range.^28,29^ Experiments on thin samples, which are thin compared to the thermal diffusion length, provide another opportunity to measure the complex heat capacity directly.^29^ Here, thin-film chip-based AC calorimetry was employed to realize experiments in a wide frequency range, particularly at high frequencies.^30,31^

In AC calorimetry, an oscillating power is applied to the sensor, and the corresponding temperature response is measured. Under quasi-adiabatic and quasi-static conditions,^32,33^ apparent heat capacity, *c*_*p*_, is given by eq 7.
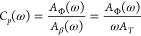
7where *A*_Φ_(ω) is the amplitude of the periodic heat flow and *A*_T_ is the temperature amplitude. The product ω*A*_T_, is the scanning rate amplitude, *A*_ß_, which also provides the limits for the measurement at low and high frequencies. For AC calorimetry, the low-frequency limit is defined by the violation of the condition of quasi-adiabaticity and the corresponding decrease in the signal-to-noise ratio.^32,33^ The heating rate amplitude, *A*_ß_, becomes immediately very large at high frequencies and not too small temperature oscillations. For *A*_T_ = 0.01 K and ω = 10^6^ rad s^–1^ (frequency, *f* = 160 kHz), the heating rate amplitude is *A*_β_ = 10,000 K s^–1^, which means the calorimeter must be able to heat and, more importantly, to cool the sample at a rate of 10,000 K s^–1^. Such high cooling rates are accessible by fast scanning chip calorimeters.^34−36^ By minimizing sample and addenda heat capacity (nJ K^–1^) and utilizing a gas as the cooling medium fast cooling can be realized.^37^ The thin-film limit for heat capacity determination is satisfied using thin (<μm) samples.^28,29^ Therefore, the inherent problems of methods measuring effusivity,^28,38^ like the 3ω-method,^21,22^ transient grating techniques,^39^ or other high-frequency thermal techniques, are not significant here.^24−26,40^

**Figure 2:**
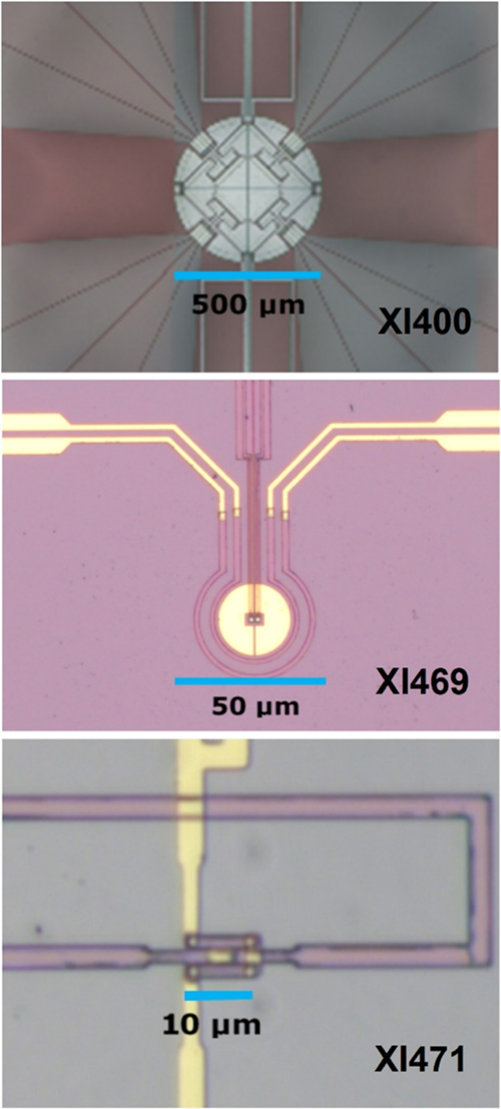
Microphotographs of the employed sensors in order of increasing frequency: XI400, XI469, and XI471. Note the different scale bars.

The employed differential AC chip calorimeter (Figure 2) with pJ K^–1^ sensitivity with a small total heat capacity (addenda + sample) and the possibility to cool fast, not only high sensitivity is achieved but also AC measurements at relatively high frequencies are possible.^41^ The calorimeter allows measurements in the frequency range 10^–2^ rad s^–1^ to above 10^6^ rad s^–1^.^30,42−44^ The basic equations for such a differential setup based on thin-film sensors are discussed in detail in refs (30, 42, 43).^30,42,43^

The PEMA sample was placed on one of the sensors for the differential AC calorimetric measurements while the reference sensor was empty.^30,31,43,44^ For the measurements covering the frequency range 10^–2^ rad s^–1^ < ω < 2 × 10^6^ rad s^–1^, different chip-nanocalorimeters from Xensor Integrations, NL, were used:^45^ Sensors XI400 (6 thermocouples), XI469 (2 thermocouples, smaller hotspot) both on ceramic housing, and XI471 (1 thermocouple) on TO5-housing.

**Figure 3:**
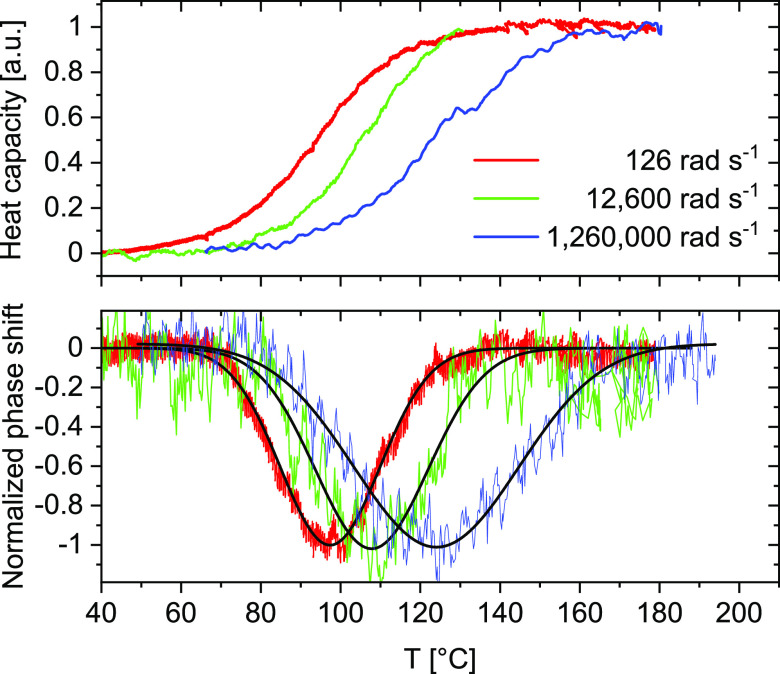
Real part of complex heat capacity and corrected phase angle for PEMA at the indicated angular frequencies.

The periodic heating is generated by the on-chip resistive heater in direct contact with the sensor membrane. The small sample in the ng range is placed as a sub-micron thin film on the very center of the sensor to access the high-frequency range. The calorimetric chips are placed into a thermostat inside a metal tube. The thermostat environment is cooled to 273 K by placing the tube inside a liquid nitrogen dewar.^30^ AC calorimetric temperature scans at 1 K min^–1^ underlying heating rate and constant frequency were performed in the vicinity of the glass transition by controlling the temperature of the thermostat. The amplitude of the thermocouple signal shows a step at the glass transition and the phase angle between power and temperature oscillation at a peak. All measured heat capacity curves, examples shown in Figure 3 for PEMA, are normalized by subtracting the tangent below and dividing by the tangent above the dynamic glass transition, to have a direct comparison of the temperature shift and the profile of the measured signals. The phase angle was corrected for the influence of the changing heat capacity at the glass transition,^46^ and normalized by the maximum value. An example of the resulting curves is shown in Figure 3.

The width of the transition interval, 2δ*T*, as needed for the calculation of the corresponding length scale, is obtained as the width of the peak in the phase angle. The Gauss fit functions, which for polymers fit quite well to the imaginary part or the phase angle or the temperature derivative of the real part versus temperature, are shown as black lines in Figure 3. The extremum of the phase angle or *c*_p_″ coincides, as usual, with the half-step temperature of *c*_p_′. As for other compliances, the position of both quantities depends on the measurement frequency and is commonly described by the Vogel–Fulcher–Tammann–Hesse (VFTH) function; see Figure 5 and Supporting Information SI1.

### C Quasi-Elastic Neutron Scattering (QENS)

The major part of the quasi-elastic neutron scattering (QENS) information used in this work was obtained from neutron spin echo (NSE) spectroscopy. From the experimental principle, this method allows access to the slowest dynamics possible with QENS—dynamics up to several hundred nanoseconds. To correct the influence of a secondary relaxation, namely that of the methyl groups, neutron backscattering spectroscopy (NBS) was used in addition, which operates at a time scale up to 2–3 ns. NSE directly produces the intermediate scattering function, which is connected with the trajectories of the atoms by^47^
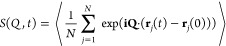
8Here, **Q** is the scattering vector associated with the momentum transfer at the scattering of the neutron by ℏ**Q** = **p′** – **p**. For small energy transfer (quasi-elastic) scattering, its norm is related to the incident wavelength, λ, and the scattering angle, θ, by *Q* = (4π/λ) sin (θ/2). In contrast, NSE measures the Fourier transform of this quantity, the scattering function
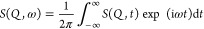
9where ℏω = Δ*E* is the energy transfer at the inelastic scattering of the neutron. In both cases, the scattering is mostly incoherent due to the large incoherent neutron cross section of hydrogen. Therefore, the self-correlation is observed. This is reflected in the summation of terms with equal indices only in eq 8.

#### 1 Neutron Spin Echo Spectroscopy

**Figure 4:**
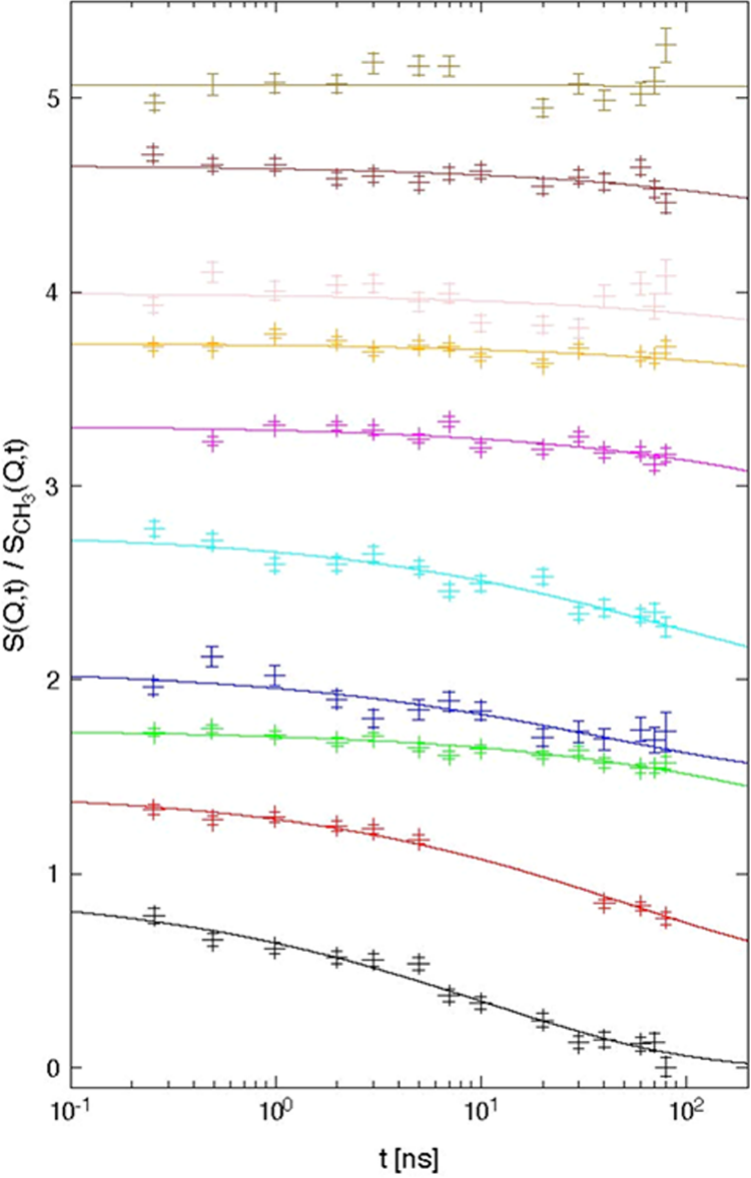
Fits of the NSE data. The NSE data are corrected for the methyl group dynamics and fitted with Kohlrausch functions with individual *τ*_K_(*Q*, *T*) and prefactors *f*_*T*_ (*Q*). The data sets/fits belong to the *T*–*Q* combinations listed in Table I (top to bottom in this figure). For clarity, data sets/fits have been offset by +0.5 with respect to the preceding one.

NSE experiments were performed on J-NSE “Phoenix” at Heinz Maier-Leibnitz Zentrum, Garching, Germany.^48,49^ Compared to the version of the instrument used in our previous work,^1^ the Fourier time range is now extended using superconducting coils for the main precession fields. Due to the fact that for spin-incoherent neutron scattering from hydrogen the performance is strongly reduced, we had to use a comparatively short neutron wavelength of 8 Å to get sufficient intensity. As a consequence, the time range was limited to 80 ns. This is a factor of 2 larger compared to 40 ns in most of the NSE spectra in ref (1). For most runs, 14 Fourier times were chosen in the range 0.25···80 ns roughly logarithmically spaced. As can be seen from Figure 4, some points had to be omitted because of interferences of the magnetic field of a neighboring instrument with the fields on the NSE. Table I shows the *T*–*Q* combinations measured in this way.

The result of the NSE method is the intermediate scattering function 7. Ideally, in the coherent scattering case, it would be normalized as *S*(*Q*, *t*)/*S*(*Q*, *t* = 0) and in the incoherent case directly be *S*(*Q*, *t*). Because the experimental integration leading to *S*(*Q*, *t* = 0) (or one in the incoherent case) is not complete, the normalization is not correct. Therefore, the NSE data, especially at the high *Q* values used here, contain a “technical factor”. To determine this factor would require additional QENS experiments at shorter times. Because a freely fitted prefactor due to fast dynamics in the sample was required anyway, no such experiments were done.

**Table I:** NSE Data Sets Included in the Evaluation

*T* [K]	*Q* [Å^–1^]	color in Figure 4
380	0.90	olive
400	0.60	brown
400	0.90	pink
420	0.20	orange
420	0.35	magenta
420	0.60	cyan
420	0.90	blue
440	0.20	green
440	0.35	red
440	0.60	black

A flat film with 0.4 mm thickness was used for the NSE experiments. In perpendicular orientation, the calculated transmission is 0.8 in agreement with the transmission measured on the instrument. Because the orientation of the sample is adjusted to half the scattering angle during the experiment, the transmission for higher *Q* measurements is smaller. The resulting high multiple scattering fraction is tolerable here because the registration of multiply scattered neutrons is strongly suppressed in a spin echo setup. The sample was placed in an aluminum container which was put into an oven to bring it to the desired temperature. The oven consists of a copper frame heated by two bifilar electrical heating cartridges (50 W each) and windows for the neutron beam made from single-crystal aluminum. At the large *Q* values used in this study, the contribution of the oven and container is small. As a background measurement, an empty aluminum container has been measured under the same conditions as the sample and properly subtracted from the measurement of the sample.

#### 2 Neutron Backscattering Spectroscopy

NBS experiments were performed on SPHERES at Heinz Maier-Leibnitz Zentrum, Garching, Germany.^50,51^ Due to the backscattering principle, the neutron wavelength is fixed by the monochromator/analyzer crystals used, here Si111, to 6.271 Å. The resolution is about 0.64 μeV (full width at half-maximum) and the accessible momentum transfer range 0.2···1.8 Å^–1^. Two types of experiments were done: (i) measuring spectra with a moving monochromator which by the Doppler effect allows to access an energy transfer range Δ*E* = −29 μeV···+29 μeV and (ii) scanning temperature with resting monochromator (Δ*E* = 0, elastic scan).

The sample used was a flat film of 0.18 mm thickness under an angle of 135°. The calculated transmission is 88% so that multiple scattering effects can be neglected. The sample was welded into an aluminum sample holder of 0.5 mm wall thickness avoiding background scattering from sealings and reducing scattering from the sample holder itself. Temperature control was realized by a closed-cycle refrigerator with an additional aluminum heat shield surrounding the sample holder to improve temperature regulation.

## IV Results

### A Neutron Scattering Spectroscopy

For the interpretation of NSE data, and QENS data in general, it has to be taken into account that these data always reflect the total dynamics of the system and not only single processes as the α relaxation. This requirement was taken into account here by considering three statistically independent processes, which leads to a product of three terms in the intermediate scattering function^47^
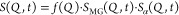
10Here, *f* (*Q*) comprises all fast (<100 ps) motions, which can be treated as a constant prefactor in the time window of the NSE experiments. It also absorbs the technical factor mentioned in Section 1. PEMA contains two methyl groups that can perform an in-place rotation even if the general structure of the molecule is rigid. This was taken into account by a model for *S*_MG_(*Q*, *t*) based on the additional backscattering measurements (see Supporting Information SI2). The data shown in Figure 4 are divided by *S*_MG_(*Q*, *t*) but still contain the factor *f* (*Q*).

The fit of the data was always done using a Kohlrausch function
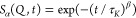
11for the α relaxation.^52^ In the first attempt, both τ_K_(*Q*, *T)* and the prefactor *f*_*T*_ (*Q*) were fitted individually for each *T*–*Q* combination. For the stretching parameter β, the same value was used for all data sets and only fitted once. In summary, the fit required 21 parameters for the 10 data sets with 132 data points in total. For this kind of specially restrained fit (and all other fits in this work), the program unifit was used,^53^ which in turn uses the subroutine VA05 from the HSL library.^54^

Figure 7 shows the resulting values for τ_K_(*Q*, *T*). It can be seen that the errors are quite large in general. Especially for *T* = 380 K, *Q* = 0.9 Å^–1^, no reliable determination of τ was possible.

To specify the characteristic scattering vector Q*, where the time scales of NSE and AC calorimetry coincide, it is necessary to interpolate (or even extrapolate) the *Q* dependence of τ_K_(*Q*, *T*). This is usually done by the empirical power law relation
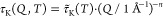
12To obtain a reliable error estimate of the final result, Q*, a different approach was used to fix this relation, which is explained in Supporting Information SI3.

### B AC Calorimetry

**Figure 5:**
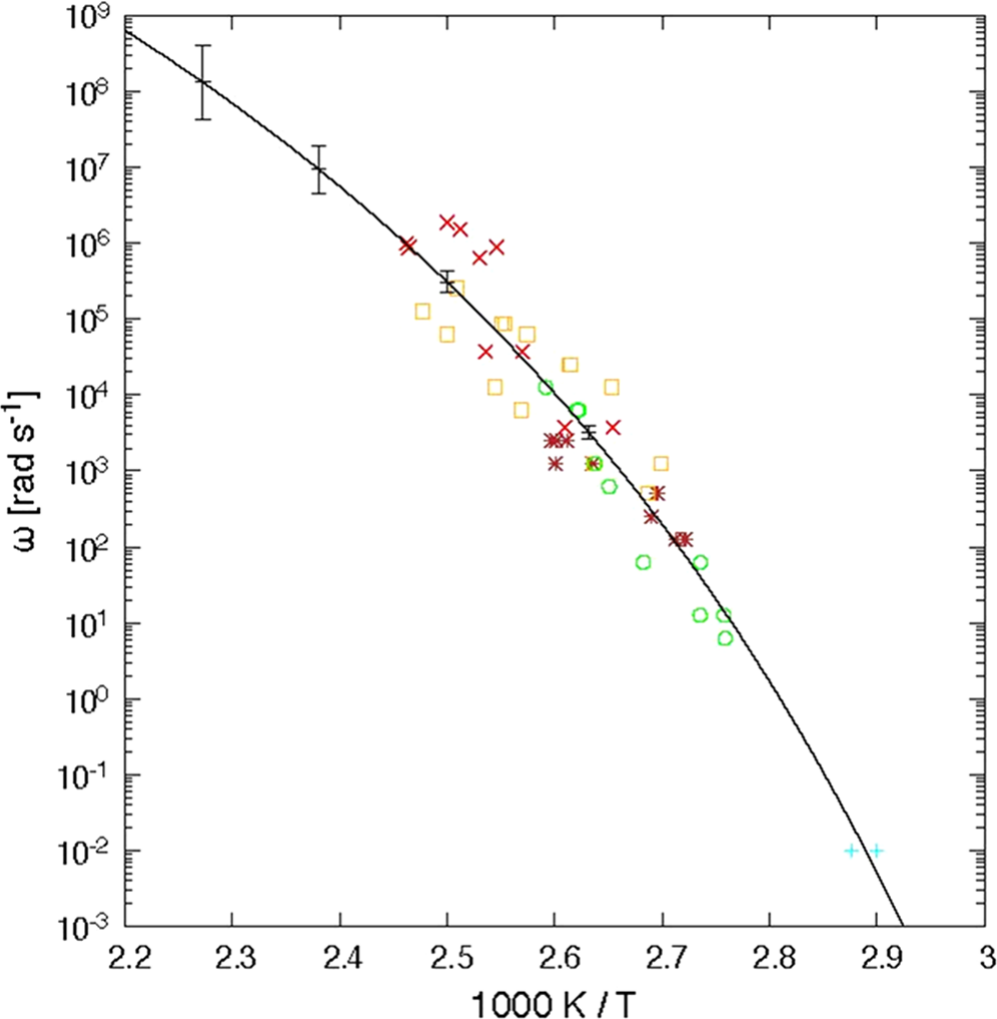
Relaxation map for PEMA. Data from different calorimetry sensors: XI471 (red crosses), XI371 (orange squares), XI400 (brown asterisks), XI469 (green circles), Pyris DSC (cyan crosses). The continuous black curve indicates the fit of ω_max_(*T*) by a Vogel–Fulcher–Tammann–Hesse relation. The extrapolated values at the temperatures of the NSE experiment (see below) are included with error bars.

The dynamic glass-transition temperatures *T*_α,dyn_ from AC calorimetry together with data from DSC are plotted in the relaxation map (Figure 5). PEMA was measured with AC calorimetry from 5 Hz to 300 kHz.

**Figure 6:**
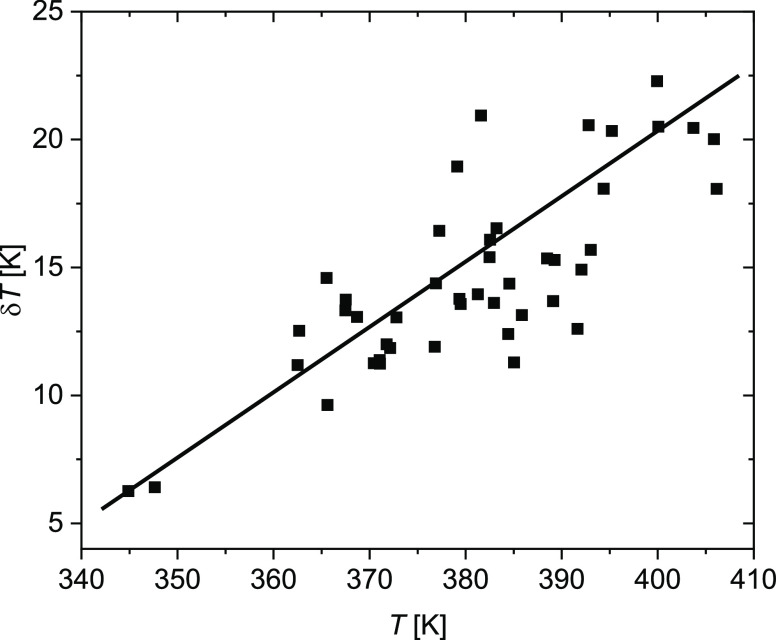
Half-width of the glass transition δ*T*, which is determined from the Gaussian fit of the phase angle curves, as shown in Figure 3. The line is a guide to the eye.

*T*_α_ was not only varying by about 60 K in the frequency range of 10^–2^ rad s^–1^ to 10^6^ rad s^–1^, but there was a substantial broadening of the dynamic glass transition with increasing frequency. The half-width of the glass transition interval (Figure 6) was obtained by fitting a Gaussian to the phase angle or the derivative of the heat capacity curves. For both fits, comparable values were obtained.

Next, the correlation length, ξ, of dynamically correlated segments at the glass transition was determined according to eq 3.

### C Combination of QENS and AC Calorimetric Data

The maximum positions in the AC calorimetric data were converted into Kohlrausch times by identifying them with the maxima of the susceptibility corresponding to the Kohlrausch times.^54^ This leads to the conversion τ_K_ = 0.725/ω_max_ with β = 0.473 from the joint fit of the NSE data. The error bars of β lead to an uncertainty of about 4% in this conversion, which is clearly negligible in comparison to the other errors entering the final result. Also, the β from the individual fits (0.441) would only change the denominator to 0.712.

**Figure 7:**
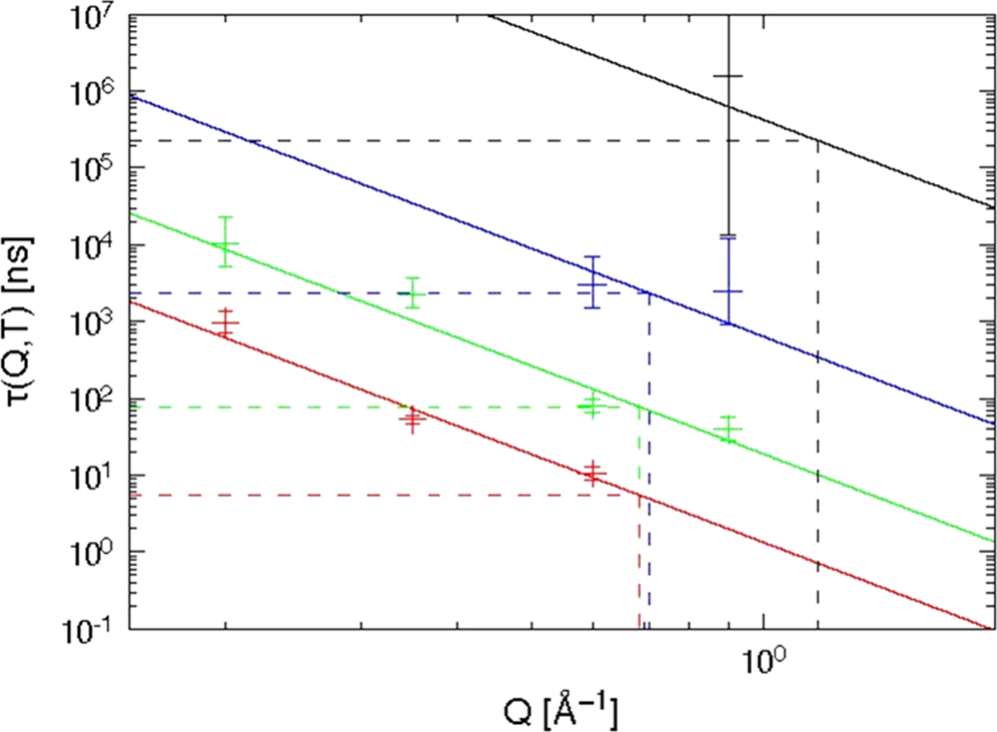
Relaxation times τ_K_(*Q*, *T*) from NSE experiments. Temperatures (bottom to top): 440 K (red), 420 K (green), 400 K (blue), 380 K (black). The errors represent 68.3% confidence intervals from a Monte Carlo simulation (see Supporting Information SI3). For *T* = 380 K, *Q* = 0.9 Å^–1^ only a lower bound of τ_K_ can be given. The lines are fits with the relation τ_K_(*Q*) ∝ *Q*^–*n*^. As explained in Supporting Information SI3, these fits were done on the original *S*(*Q*, *t*) data and not on the τ_K_(*Q*, *T*) values.

Table II shows the resulting τ_K_^AC^ values and the Q* values obtained as the solution of
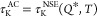
13This calculation is graphically represented in Figure 7 by the dashed lines. The confidence intervals given in the table result from the procedure described in Supporting Information SI3.

**Table II:** Determination of the Cooperativity Length from the Combination of AC Calorimetry and NSE Data.

*T* [K]	ω_max_ [rad/s]	τ_K_^AC^ [ns]	Q* [Å^–1^]	*l* [nm]
380	3.21 (2.62···3.94) ·10^3^	2.26 (1.84···2.77) ·10^5^	1.18 (0.39···∞)	0.379 (0···1.147)
400	3.08 (2.21···4.29) ·10^5^	2.36 (1.69···3.28) ·10^3^	0.71 (0.57···90)	0.630 (0.497···0.785)
420	9.29 (4.53···19.1) ·10^6^	78.0 (38.0···160)	0.69 (0.50···0.96)	0.649 (0.466···0.894)
440	1.31 (0.42···4.10) ·10^8^	5.55 (1.77···17.4)	0.69 (0.41···1.17)	0.650 (0.382···1.091)

ω_max_ is the maximum position in the imaginary part of the dynamic heat capacity. τ^AC^ is the equivalent Kohlrausch time calculated as *τ*_K_ = 0.725/ω_max_. Q* is the scattering vector at which the Kohlrausch time of the NSE experiment coincides with the latter. *l* is the cooperativity length calculated as spherical diameter. The range in parentheses denotes the confidence interval of the respective parameter.

As in ref (1), the Q* values were converted into cooperativity lengths by the relation  resulting from the assumption of spherical CRRs. Table II lists the calculated values together with the intervals in which the actual value is expected to lie with 68% probability. This interval corresponds to that spanned by the standard error in the case of a normal-distributed parameter. But it can be seen that the intervals are asymmetric and very wide here so that conventional error propagation based on a normal distribution is not applicable. Especially, because the NSE data only give a lower limit of Q* for 380 K in that case, only an upper limit of *l* can be predicted.

## V Discussion

**Figure 8:**
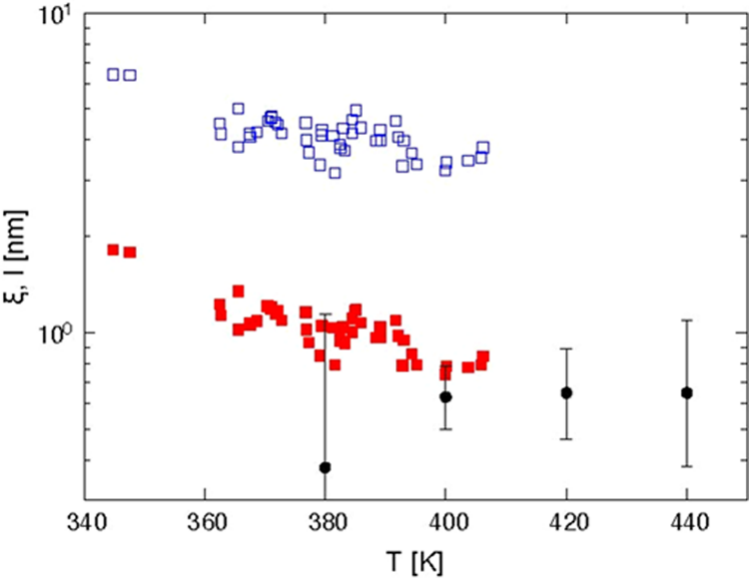
Comparison of the characteristic length ξ_α_ from thermodynamic arguments with *l* from the combination of AC calorimetry and NSE: ξ_α_ without considering temperature fluctuations (blue open squares), ξ_α_ accounting for temperature fluctuations (red filled squares), *l* from AC calorimetry and NSE (black circles).

Figure 8 shows the comparison of the “thermodynamic” derivation of the characteristic length ξ_α_ by eqs 1–3 with the cooperativity length *l* from the combination of AC calorimetry and NSE as described in the preceding section. It can be seen that the latter values clearly agree better with the calculation involving temperature fluctuations, eq 2.

As can be seen from the error bars, the uncertainty of the values *l* is rather large, even though standard error bars represent a rather “relaxed” confidence criterion of 68%. The reason for this is mostly that there is only a marginal overlap between the two techniques, AC calorimetry and NSE. The most reliable point in this respect at *T* = 400 K deviates by 20% from its ξ_α_(δ*T ≠* 0) counterpart. This could be marginally explained by errors due to extrapolation and statistical uncertainty. Also from the trend in the calorimetric values, one would expect still some decrease of the length in the temperature range 400···440 K. This is not seen in the nominal *l* values from AC/NSE although possible within their error bounds. In the following, some conceptual and systematic sources for deviations will be considered.

The procedure described in ref (55) does not include exact definitions of some involved quantities. For example, for δ*T* and δ*T*_g_, the quantitative definition of “δ” is missing. It is common practice to use the half-width at half-maximum of the peak in the temperature-dependent specific heat at a certain frequency. But alternatively, also the square root of the variance of a fitted Gaussian could be used. This would give a value slightly lower by the factor *f* = 1/(√2 ln 2 ≈ 0.85). In consequence, *N*_α_ would increase by a factor *f*^–2^ and ξ_α_ by *f*^–2*/*3^ = 1.12. This would result in a small change only and even in the wrong direction.

Similarly, because the shape of the cooperatively rearranging regions (CRR) is not predicted, relation 3 can be doubted. Taking ξ_α_ as the edge length of a cube instead of the diameter of a sphere would change it by a factor 0.81. This change would resolve the discrepancy but a cubic CRR is a rather artificial construct.

The conversion of the dynamic coincidence wave vector Q* to a length *l* is also connected with some uncertainties. Since scattering functions in reciprocal space are related to densities in real space by a Fourier transform, there is no one-to-one relation between *Q* values and lengths. Motivated by the periodicity of exp(i*Qr*), often *l* ≈ 2*π*/*Q* is used. But that is highly dubious here because *r* refers to a distance in space but *l* to the size of an object.

The conversion done here is based on the assumption that the length is the diameter of a sphere whose radius of gyration appears in the intermediate scattering function.^1^ Although this is a reasonable assumption, it is by no means compelling.

It also should be noted that for a change of the assumed shape of the CRR also the shape assumed in this calculation has to be replaced. For example, for a cube, one would obtain .

So for this particular change, the *l* values would decrease by a factor 0.77. This is close to the same relative change of ξ_α_. The discrepancy as the ratio ξ_α_/*l* would even increase by 4%. Again it may be possible to conceive particular shapes with the effect of reducing that discrepancy, but it seems unlikely to obtain a close match in that way.

Finally, a similar problem occurs in the combination of frequency-dependent (AC calorimetry) and time-dependent (NSE) measurements. Relation 6 results from the maximum of a fictitious susceptibility derived from the (Kohlrausch) fit function of the NSE data. Again this is a motivation but not a compelling argument. Even a simple conversion as *τ* = 1/ω cannot be rejected immediately. It would lead to a factor of 1.38. But because the latter calculation implies *l* ∝ *τ*^1/*n*^, the change of *l* would be by a factor 1.09 leading to a slightly better agreement.

In summary, the discrepancy between the cooperativity length ξ_α_(δ*T* ≠ 0) derived from thermodynamics assuming temperature fluctuations and the length *l* obtained from AC calorimetry and NSE lies in the range which can be explained by statistical errors in the data but also in the order of methodological uncertainties. The nominal value of *l* at 380 K deviates more strongly but still agrees within error bars. On the other hand, the discrepancy to the thermodynamical calculation without temperature fluctuations, ξ_α_(δ*T* = 0), is a factor of about 10 and certainly significant.

## VI Conclusions

The present study follows the strategy outlined in the preceding publication:^1^

“To obtain more definite answers regarding the existence of temperature fluctuations in nanoscale subsystems, the approaches as presented in ref (1) by comparing the length scales from AC calorimetry and QENS should be continued with the guidelines: (i) A material should be chosen where the difference between the cooperativity values considering energy and temperature fluctuations (eqs 1 and 2) is larger. (ii) The material must not have any disturbing relaxation in the range of the α-relaxation in the QENS experiments as propylene glycol. (iii) The overlap of temperatures and time scales should be large”.

With PEMA and the further developed experimental techniques, all three objectives were now fulfilled. The overlap of temperatures and time scales (iii) was sufficient, and there was no disturbing additional relaxation (ii). Most importantly, a ratio of 4, instead of 2 as in the preceding study,^1^ is expected for the determination of the characteristic length considering or neglecting temperature fluctuations (i).

The new data on PEMA are in agreement with ref (1) and allow the following conclusions: (i) At the same temperature of 400 K and the same time scale of τ_K_ of ca. 2 μs, both independent methods obtained with AC calorimetry and QENS yield characteristic length scales close to 1 nm. By this, the existence of a characteristic length scale for the cooperative motions relevant to the glass transition is again supported. (ii) Furthermore, since the estimate of this characteristic length scale considering temperature fluctuations provides a much better match (about a factor of 4) between the length scales from AC calorimetry and QENS, the existence of temperature fluctuations in nanoscale subsystems is supported.

Although the basic aim of an overlap between QENS and AC calorimetry has been achieved, there is room for improvement. Since the factor of 4 between the thermodynamic calculations of *N*_α_ is difficult to surpass without resorting to more complex materials tending to have additional secondary relaxations, such an improvement would be rather due to experimental advances. For NSE, future high-intensity neutron sources (e.g., ESS) may offer the possibility to construct instruments with a larger time range.^56^ On the side of AC calorimetry, higher frequencies up to 10^8^ Hz seem feasible,^24−26,40^ even new challenges appear, yielding a wider overlap range between AC calorimetry and QENS. In addition, molecular dynamics simulations may play an important role in the identification of *N*_α_ in suitable model systems or even atomistically realistic simulations.

## Data Availability

The data that support the findings of this study are available from the corresponding author upon reasonable request.

## Supporting Information Available

Extrapolation of dynamic calorimetry to temperatures ≥400 K with error calculation (SI1); calculation of the correction of NSE data for dynamics resulting from methyl group rotation (SI2); and description of the fit procedure to derive *Q** from NSE with Monte Carlo error estimation (SI3) (PDF)
