# Supplementary material for: Determination of Cooperativity Length in a Glass-Forming Polymer
Source: ACS Phys Chem Au. 2023 Jan 4;3(2):172–80. doi: 10.1021/acsphyschemau.2c00057 (PMC10037462; doi:10.1021/acsphyschemau.2c00057)
Supplement: Supplementary file 1 — pg2c00057_si_001.pdf [file pg2c00057_si_001.pdf]

Supporting Information for:

Determination of the cooperativity length in a glass-forming polymer

Yeong Zen Chua,[1,2] Reiner Zorn[*,3] Jürn W. P. Schmelzer,[1,2] Christoph Schick,[1,2] Olaf Holderer,[4] and Michaela Zamponi[4]

[1)] Institute of Physics, University of Rostock, Albert-Einstein-Str. 23-24, 18051 Rostock, Germany

[2)] Competence Centre CALOR, Faculty of Interdisciplinary Research, University of Rostock, Albert-Einstein-Str. 25, 18051 Rostock, Germany

[3)] Forschungszentrum Jülich GmbH, Jülich Centre for Neutron Science (JCNS-1) and Institute for Biological Information Processing (IBI-8), 52425 Jülich, Germany

[4)] Forschungszentrum Jülich GmbH, Jülich Centre for Neutron Science at MLZ, Garching 85748, Germany

[*] r.zorn@fz-juelich.de

(Dated: 14 December 2022)

SI1: EXTRAPOLATION OF DYNAMIC CALORIMETRY

Because there is no complete overlap between the temperature ranges of the NSE experiments and the calorimetric ones it is necessary to extrapolate the latter to higher temperatures. A description by an empirical function $\omega_{max}(T)$ also serves the purpose of reducing the influence of statistical fluctuations in the dynamic calorimetry data. Usually, the Vogel-Fulcher-Tammann-Hesse (VFTH) equation is used for this purpose:

$$\log_{10}\left(\omega_{max}(T)\right) = -A - B/(T - T_0)\,, \qquad \text{(SI1)}$$

where ω_{max} is measured in rad/s. We use this formulation in order to be compatible with.[1] It is easy to see that it is equivalent to the standard expression, $\omega_\infty \exp(-B'/(T - T_0))$. Fitting the logarithm instead of the value of the maximum frequency takes account of the fact that the errors in ω_{max} are rather constant relative errors than constant absolute ones. The fit

results in the function which is indicated in Figure 5 of the main text as continuous curve. The fit parameters are $A = -17.2 \pm 3.5$, $B = 1620$ K ± 764 K, and $T_0 = 261.7$ K ± 25.8 K. Although the resulting A value is exceptionally high, we consider this fit useful for the extrapolation by just 20 ... 40 K. The extrapolated values of $\omega_{\max}$ were calculated by (SI1) from these parameters and are shown in Table III.

TABLE III. Extrapolation of calorimetric data to NSE temperatures. The central column shows the extrapolated value of the logarithmic frequency obtained by fitting equation (SI2). The right column gives the extrapolated frequency itself. The asymmetric error bars are a consequence of the large error in the logarithm together with the non-linearity of the power function.

T_1 [K]	$\log_{10} \omega_{\max}(T_1)$	$\omega_{\max}(T_1)$ [rad/s]
380	3.507 ± 0.089	$3.21 \left\{ \begin{matrix} +0.73 \\ -0.60 \end{matrix} \right\} \cdot 10^3$
400	5.488 ± 0.144	$3.08 \left\{ \begin{matrix} +1.21 \\ -0.87 \end{matrix} \right\} \cdot 10^5$
420	6.968 ± 0.312	$9.29 \left\{ \begin{matrix} +9.76 \\ -4.76 \end{matrix} \right\} \cdot 10^6$
440	8.116 ± 0.497	$1.31 \left\{ \begin{matrix} +2.80 \\ -0.89 \end{matrix} \right\} \cdot 10^8$

For judging the validity of the conclusions it is important to know the confidence intervals of the extrapolated values. This is not possible by a straightforward error propagation from the fit parameters. Because the fit parameters are strongly correlated the actual error bars are much smaller than the ones expected from error propagation. Therefore, we modified the fit function such that instead of A the logarithm of the extrapolated value at the desired temperature, $x = \log_{10}(\omega_{\max}(T_1))$, is the fit parameter:

$$\log_{10}(\omega_{\max}(T)) = x + B/(T_1 - T_0) - B/(T - T_0) \,. \qquad \text{(SI2)}$$

With this change, standard fit routines with estimation of confidence intervals give the error bar of the extrapolated value $\log_{10}(\omega_{\max}(T_1))$ directly. (It is easy to see that the values and error bars of the remaining parameters B and T_0 are unchanged by the substitution of A.) The

error bars are included in Table III and Figure 5 of the main text. It can be seen that they increase strongly when the region of measured data is left. They are small only for 380 K and 400 K where the ‘extrapolation’ is more like an averaging of neighboring data points. For the other temperatures the extrapolation leads to large errors which do not make an interpretation of the NSE data impossible but introduce larger errors than those of the NSE experiment itself.

SI2: CORRECTION OF THE NSE DATA FOR METHYL GROUP ROTATION DYNAMICS

The first successful experiment of this kind was performed on propyleneglycol (PG).[1] One of the complications which became clear during the evaluation was that PG contains a methyl group (MG) which can rotate already at comparatively low temperatures. For most of the temperatures the dynamics of the MG was already ‘relaxed-out’ on the time scale of NSE. There, the MG dynamics just appears as a Debye-Waller factor which is absorbed in the general prefactor necessary to fit the NSE data. Nevertheless, at the lowest temperatures the MG dynamics and the α relaxation were overlapping and had to be disentangled. Fortunately, the exact shape and temperature dependence of the MG dynamics were known from another experiment.[2]

For the experiment here, one reason to choose PEMA was its higher T_g. This feature shifts the experiment temperatures into a range where the relaxation of the two MGs per monomeric unit are far above where one would expect the MG dynamics to be visible on NSE. Nevertheless, an additional quasielastic neutron scattering experiment was done on the neutron backscattering instrument SPHERES (MLZ, Garching) to determine the parameters of the MG dynamics.

Because a full study of the MG dynamics was not intended only an elastic scan, two spectra at temperatures where the MG dynamics is expected to fall into SPHERES’ dynamic range, and a low temperature spectrum as resolution function were measured. No additional time-of-flight neutron spectroscopy experiments were done and usually there are more than two temperatures measured to obtain a solid basis to establish the temperature dependence. Nevertheless, it was possible to derive the full set of parameters describing the MG dynamics in the rotation rate distribution model (RRDM).[3, 4] Literature data on the methyl group rotation in PEMA exists,[5] but because the authors used a side-group-deuterated PEMA it covers only one of the two methyl groups per monomeric unit. The two methyl groups are chemically different and therefore expected to have different dynamics.

The backscattering data were reduced using the program SQW for the spectra and its variant SQWel for the elastic scans.[6] For the spectra, the result is the scattering function $S(Q, \omega)$ convoluted with the resolution function $R(Q, \omega)$:

$$\tilde{S}(Q,\omega) = \int S(Q,\omega')R(Q,\omega - \omega')d\omega'. \tag{SI3}$$

(The boundaries of the integral are $-E_{\mathrm{inc}}/\hbar$ with E_{inc} being the incident energy of the neutrons and $+\infty$. The convolution is also just an approximation which gets worse for $|\hbar\omega| \approx E_{\mathrm{inc}}$. These concerns are not relevant here because the energy range of SPHERES, ±29 μeV, is much smaller than E_{inc} = 2.08 meV.) The resolution function itself was determined as the spectrum at a sufficiently low temperature (3.5 K) where it is assumed that all motions are frozen, $S_{\mathrm{low}\ T}(Q, \omega) = \delta(\omega)$, thus:

$$R(Q, \omega) = S_{\mathrm{lowT}}(Q, \omega)\ . \tag{SI4}$$

The elastic scan represents the value of $S(Q, \omega = 0)$ at varying temperature:

$$S_{\mathrm{el}}(Q,T) = \int S_T(Q,\omega')R(Q,-\omega')d\omega'. \tag{SI5}$$

For methyl group dynamics, the scattering function is given by

$$S(Q, \omega) = (1 - f_{\mathrm{mob}})\ \delta(\omega) + f_{\mathrm{mob}}\ (A_0(Q) + (1 - A_0(Q))\varphi(\omega)) \tag{SI6}$$

Here f_{mob} is the fraction of hydrogen atoms mobile in methyl groups,[7] 6/10.

The elastic incoherent structure factor (EISF) of the methyl group dynamics is that of a three-fold rotation,

$$A_0 = \frac{1}{3} + \frac{2}{3}\left(1 + \frac{\sin Qd_{HH}}{Qd_{HH}}\right), \tag{SI7}$$

where d_{HH} = 1.78 Å is the distance between H atom positions in the methyl group. Finally, in the RRDM, the relaxation function is given by a distribution of individual Debye relaxations

$$\Phi(\omega) = \int_0^\infty \frac{1}{\pi} \frac{\tau_{MG}^{-1}}{\omega^2 + \tau_{MG}^{-2}} g(E_{\mathrm{A}})\mathrm{d}E_A \tag{SI8}$$

whose relaxation times are derived by an Arrhenius law

$$\tau_{\mathrm{MG}} = \tau_{\mathrm{MG}}^{\infty}\exp\left(E_{\mathrm{A}}/k_{\mathrm{B}}T\right) \tag{SI9}$$

from a normal distribution of energy barriers,

$$g(E_A) = \frac{1}{\sqrt{\Delta E_A}} \exp\left(-\frac{(E_A - E_A^0)^2}{2\Delta E_A{}^2}\right). \tag{SI10}$$

Of course, the methyl group dynamics does not completely describe the quasielastic scattering even at the temperatures where the polymer specific relaxations (α, β ...) are not active in the window of QENS. There is also a significant amount of fast dynamics in the picosecond range already present a lowest temperatures.[8] For the spectra this would not be a significant complication because on the dynamic scale of backscattering these just represent a Debye-Waller-like constant factor (DWF). But because this factor is temperature dependent, for the elastic scans it has to be considered more thoroughly. Simplifying, we assume that this dynamics is vibrational and resulting from a Debye-model density of states. In that case the DWF is given by:

$$\mathrm{DWF}(Q,T) = \exp\left(-\frac{3\,\hbar Q^2}{2M\omega_D}\left(2\frac{k_B T}{\hbar\omega_D} D_1\left(\frac{\hbar\omega_D}{k_B T}\right) + \frac{1}{2}\right)\right) \tag{SI11}$$

where M is the effective mass of the scattering atoms (assumed to be the average mass of the atoms in PEMA, 6.34 amu), ω_D the Debye frequency (related to the Debye temperature by $\theta_D = \hbar\omega_D/k_B$), and $D_1(x)$ is the first Debye function,

$$D_1(x) = \frac{1}{x}\int_0^x \frac{t}{e^t - 1}\,dt. \tag{SI12}$$

The actual fits were done for the spectra with the product of (SI11) and (SI3), and for the elastic scans with the product of (SI11) and (SI5). In principle, the functions are able to fit all Q values, but in order to avoid an influence of multiple scattering only the highest two values were used, 1.42 and 1.71 Å^{-1}. In total 4402 data points were fitted with six fit parameters: θ_D, $\tau_{MG}{}^{\infty}$, E^0 , ΔE_A, and two amplitude factors for the scans at the two Q values.

The fit procedure is rather time consuming because the fit functions are nested integrals. The integral in equation (SI8) was approximated by a summation of 200 E_A points chosen such that the values of the cumulative distribution function are equidistant. The integrals (SI3) and (SI5) were calculated by the subroutine QA05 from the HSL library using an adaptive scheme based on Romberg extrapolation and trapezoidal rule.[9] The total time to carry out 77 function calls leading to a satisfactory convergence was 45 minutes on a 2.8 GHz Intel Core i7 processor using 8 parallel threads.

Because the model does not contain a Q dependence of τ_{MG} and EISF and DWF are also varying only weakly with Q, detectors were strongly grouped to obtain two averaged Q values,

$Q = 1.42$ Å^{-1} and $Q = 1.71$ Å^{-1}. Figure 1 shows the fits and Table IV the resulting parameters. It can be seen that there is a significant difference to the parameters from.[5] Especially, the average activation E^0 energy is significantly lower. This shows that the side-group methyl group is obviously more mobile than the chain-bound. But it also has to be taken into account that the fit here has slightly different conditions because the amplitudes are related by expression (SI11) while in Ref. [5] they were presumably free for each temperature.

Concerning the Debye temperature it is possible that it is influenced by a correlation with the methyl group dynamics parameters in the fit. Nevertheless it is equal to the geometric mean of the Debye temperatures given in,[10] $\theta_1 = 622$ K and $\theta_3 = 60$ K. In that case the Debye temperatures were derived by the Tarasov model.[11, 12] This model combines a one-dimensional Debye model with the usual 3D one. Because the model is expressed in terms of the heat capacity it is not immediately clear how to construct the DWF for the 1D part.

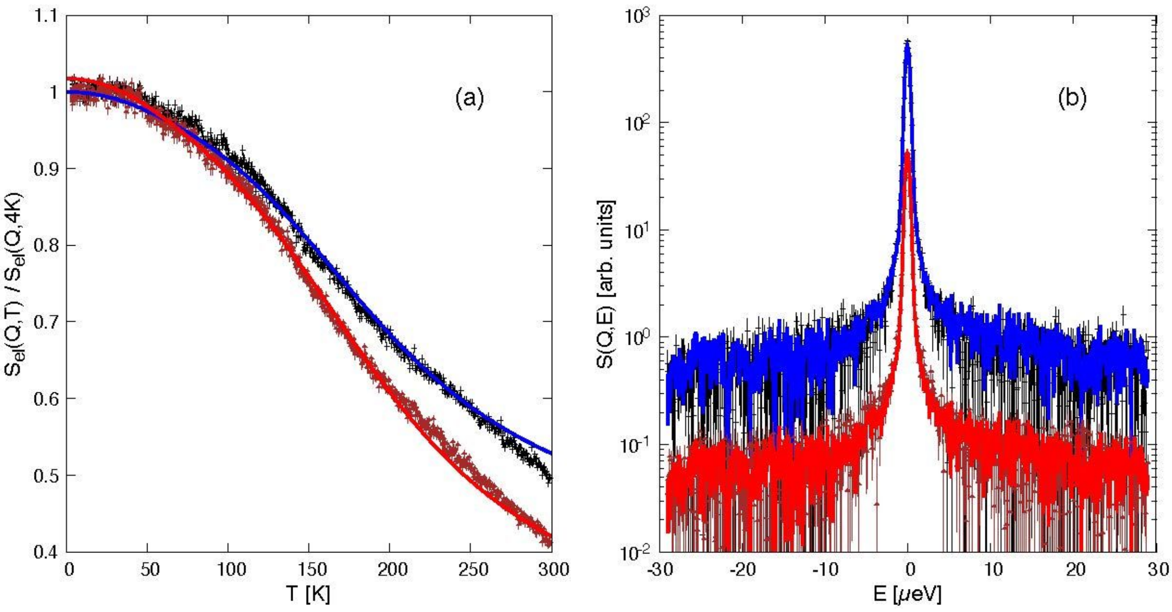

Figure 1 Fits of the backscattering data for the methyl group dynamics. (a) Fit of the elastic scans at $Q = 1.42$ Å^{-1} (black/blue) and $Q = 1.71$ Å^{-1} (brown/red). (b) Fits of the spectra at 130 K (black/blue) and 160 K and $Q = 1.71$ Å^{-1} (brown/red, offset by ×0.1). The fluctuations in the fit function are due to the fact that in equation (SI3) the real experimental function at low temperature was used as resolution and not a smoothed curve. Because a large part of the scattering is concentrated in the delta function of (SI6) the statistical fluctuation are reproduced. Therefore, this literature data could not be used to avoid the additional fit parameter.

TABLE IV. Parameters determined for the RRDM description of the methyl group dynamics in PEMA in comparison to those from Ref. [5] for the chain-bound methyl group.

Parameter	Value from scans and spectra	Ref. [5]
θ_D [K]	194.9 ± 0.9	—
τ_{MG}^{∞} [ps]	0.8 ± 0.3	0.21
E_A^0/k_B [K]	1514 ± 63	2282
$\Delta E_A/k_B$ [K]	428 ± 25	484

For the correction of the NSE data the inverse Fourier transforms of (SI6) and (SI8) are used. These are

$$S(Q,\ t) = (1 - f_{mob}) + f_{mob}\ (A_0(Q) + (1 - A_0(Q))\varphi(t)) \qquad \text{(SI13)}$$

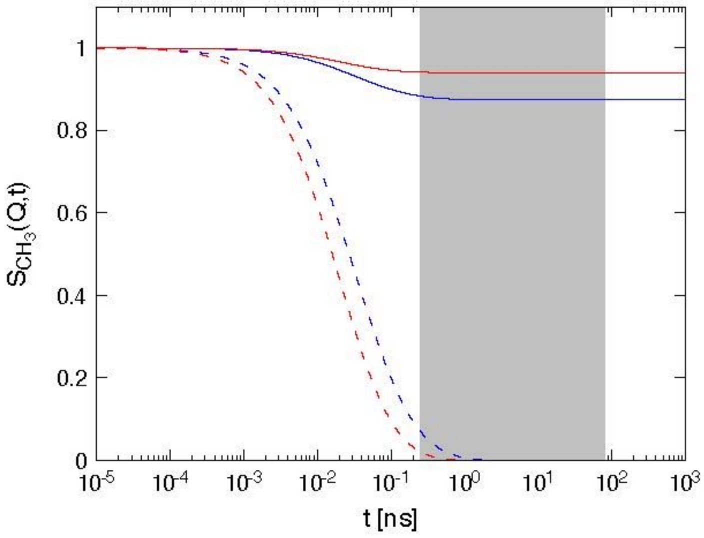

Figure 2 Methyl group dynamics of PEMA. The dashed curves show the relaxation function (SI14) of the methyl groups and the continuous curves the intermediate scattering function (SI13) of the methyl groups only. The blue and red curves represent the extremal parameters of the NSE experiments $T = 380$ K, $Q = 0.9$ Å^{-1} and $T = 440$ K, $Q = 0.6$ Å^{-1}. All other NSE experiments fall between these two. The grey region is the time domain in which NSE experiments were carried out.

and

$$\varphi(t) = \int_0^\infty \exp\left(-t/\tau_{\mathrm{MG}}\right) g(E_\mathrm{A})\, dE_\mathrm{A}, \tag{SI14}$$

respectively. The result is plotted in Figure 2 for the two extreme T/Q combinations of the NSE experiments. The continuous curves show the function by which the NSE data were divided to correct for the methyl group dynamics. It can be seen that the strategy choosing a higher T_g material was successful. The methyl group dynamics is nearly 'flat' in the NSE window and this constant factor is absorbed in the prefactor used in the NSE fitting. So the correction (albeit actually carried out) was negligible in the time range of the NSE experiments.

SI3: FIT OF THE NSE DATA TO OBTAIN *Q**

From the NSE data it is immediately clear that a fit is not easy. The data have comparatively large uncertainty and in some of the data sets the decay is weak over the times observed. By coupling the β values in (10) the fit was stabilized sufficiently to converge in an unambiguous way. (A coupling of $f(Q)$ was not possible because there is no independent knowledge on its Q and T dependence.) Nevertheless, the standard error calculation based on the inversion of the Hessian matrix gives unrealistic values. The obvious reason is that the dependence of the fit functions on τ_K is highly non-linear. Therefore, we used a Monte Carlo simulation based on 1000 fictitious data sets created with the real errors around the fit function values.[13] The values in the last column of Table V and the error bars shown in Figure 7 of the main text represent the confidence intervals in which 68.3% of the fit result lie. Only if the 'classical' errors are significantly smaller than the value itself these agree roughly with the interval spanned by the conventional error estimates. The result for 380 K/0.9 Å^{-1} shows that 14 μs is a lower bound for τ_K. The upper limit of $3.5 \cdot 10^{16}$ ns is clearly meaningless. It results from the fact that above a certain value of τ_K, within numerical precision $S(Q, t)$ = const and the fits produce arbitrary final values of the iteration. For that reason, even a complete arrest cannot be excluded. Therefore, the upper limit of τ_K(380 K) was replaced by ∞ in the following calculations. The common value of the stretching exponent was determined as $\beta = 0.441$ with a 68.3%-confidence interval of 0.404 ... 0.486.

TABLE V. Results of the *individual* fits of the NSE data. “Classical error” denotes the error calculated based on the inversion of the Hessian matrix. Confidence intervals were determined as the intervals in which 68.3% of the fit results of 1000 simulated data sets lie.

T [K]	Q [Å^{-1}]	Fit value τ_K [ns]	‘classical’ error of τ_K [ns]	MC confidence interval of τ_K [ns]
380	0.90	$1.6 \cdot 10^6$	$3.6 \cdot 10^7$	$1.4 \cdot 10^4 \ldots 3.5 \cdot 10^{16}$
400	0.60	2983	3219	1535…6919
400	0.90	2468	4353	925…12019
420	0.20	10250	11100	5132…23635
420	0.35	2293	1503	1507…3655
420	0.60	81	23	68…98
420	0.90	40	20	28…56
440	0.20	982	441	733…1341
440	0.35	53	10	46…61
440	0.60	10.5	3.1	8.5…12.7

TABLE VI. Results of the fits of the NSE data *assuming a power law* $\tau_K(Q) \propto Q^{-n}$. $\tilde{\tau}_K$ is the value of that power law at $Q = 1$ Å^{-1}. Confidence intervals were determined as the intervals in which 68.3% of the fit results of 1000 simulated data sets lie.

Parameter	Value	Confidence interval
n	3.81	3.50…4.30
β	0.473	0.409…0.522
$\tilde{\tau}_K$(380 K) [ns]	$4.2 \cdot 10^5$	$3.1 \cdot 10^3 \ldots \infty$
$\tilde{\tau}_K$(400 K) [ns]	642	281…2046

$\tilde{\tau}_{\mathrm{K}}(420\ \mathrm{K})$ [ns]	19.0	13.3…24.5
$\tilde{\tau}_{\mathrm{K}}(440\ \mathrm{K})$ [ns]	1.35	0.80…1.84

In the preceding work we used values as in Table V to fit the power law (11).[1] This is problematic here because of the weighting of the individual $\tau_{\mathrm{K}}(Q, T)$ values in such a fit. It is clear that, e.g., the values from 400 K should have less influence on the exponent of the power law than the more precise values at higher temperatures. If the errors were small, a weighting with $\Delta\tau_{\mathrm{K}}(Q, T)^{-2}$ from the conventional error estimates would be acceptable. Clearly, this is not the case here. Therefore, we fitted all $S(Q, t)$ data directly with the combined fit function

$$S_\alpha(Q,t) = \exp\left(-\left({t}/\left(\tilde{\tau}_{\mathrm{K}}(T)\cdot\left(Q/1\text{Å}^{-1}\right)^{-n}\right)\right)^{\beta}\right). \qquad \text{(SI15)}$$

Instead of 10 parameters, $\tau(Q, T)$, now only four parameters, $\tilde{\tau}_{\mathrm{K}}(T)$, are necessary, which also stabilizes the fit. The results of the fit are shown in Table VI with the confidence intervals determined as before by a Monte Carlo simulation. In Figure 7 of the main text it can be seen that the power law represents the actual fit values well. Again, for 380 K only a lower bound of $\tilde{\tau}_K$ can be obtained.

As in the case of the extrapolation of the dynamic calorimetry data, also here the problem arises that the confidence intervals derived are those of the parameters of the interpolating function (C1) but what we rather want to know are the confidence intervals in Q^*. Therefore we rewrite this function such that Q^* is a fit parameter and the τ^{AC} values to be matched are inserted:

$$S_\alpha(Q,t) = \exp\left(-\left({t}/\left(\tau_{\mathrm{K}}^{\mathrm{AC}}(T)\cdot\left(Q/Q^*\right)^{-n}\right)\right)^{\beta}\right). \qquad \text{(SI16)}$$

From the fit with this function the values in Table VII are obtained with confidence intervals. Because the function is not changed with respect to the parameters β and n these are the same as in Table VI. For 380 K, only a lower bound on Q^* can be stated.

TABLE VII. Q^* values from the fits of the NSE data with function (C2). Confidence intervals were determined as the intervals in which 68.3% of the fit results lie. The first confidence interval is that resulting from the uncertainty in the NSE data only, the second from the AC data, and the third is the compound one resulting from both uncertainties.

T [K]	Q^* [Å^{-1}]	C. i. NSE [Å^{-1}]	C. i. AC [Å^{-1}]	C. i. total [Å^{-1}]
380	1.18	0.39…∞	1.12…1.25	0.39…∞
400	0.71	0.61…0.86	0.65…0.77	0.57…0.90
420	0.69	0.66…0.72	0.57…0.83	0.50…0.96
440	0.69	0.65…0.73	0.51…0.93	0.41…1.17

The confidence intervals calculated so far do not include the uncertainty in τ^{AC} resulting from the extrapolation of $\omega_{max}(T)$. By error propagation the influence of these errors on Q^* can be calculated:

$$\frac{\Delta Q^*}{Q^*} = \Delta \ln Q^* = \frac{\ln 10}{n} \Delta \log_{10} \omega_{max}. \qquad \text{(SI17)}$$

The resulting confidence intervals are shown in the fifth column of Table VII. This table shows the dilemma of this experiment: At high temperatures (420 K, 440 K) the values from NSE are reliable but the AC data need a lot of extrapolation. At low temperatures (380 K, 400 K) the AC data do not need extrapolation but the NSE data introduce large errors.

REFERENCES

(1) Chua, Y. Z.; Zorn, R.; Holderer, O.; Schmelzer, J. W. P.; Schick, C.; Donth, E. Temperature fluctuations and the thermodynamic determination of the cooperativity length in glass forming liquids. *J. Chem. Phys.* **2017**, *146* (10), 104501. DOI: http://dx.doi.org/10.1063/1.4977737.

(2) Zorn, R.; Mayorova, M.; Richter, D.; Frick, B. Inelastic neutron scattering study of a glass-forming liquid in soft confinement. *Soft Matter* **2008**, *4* (3), 522-533, 10.1039/B713465G. DOI: http://dx.doi.org/10.1039/b713465g.

(3) Chahid, A.; Alegria, A.; Colmenero, J. Methyl Group Dynamics in Poly(vinyl methyl ether). A Rotation Rate Distribution Model. *Macromolecules* **1994**, *27* (12), 3282-3288. DOI: https://doi.org/10.1021/ma00090a022.

(4) Mukhopadhyay, R.; Alegría, A.; Colmenero, J.; Frick, B. Methyl Group Dynamics in Poly(vinyl acetate): A Neutron Scattering Study. *Macromolecules* **1998**, *31* (12), 3985-3993. DOI: https://doi.org/10.1021/ma970575p.

(5) Genix, A. C.; Arbe, A.; Colmenero, J.; Wuttke, J.; Richter, D. Neutron Scattering and X-ray Investigation of the Structure and Dynamics of Poly(ethyl methacrylate). *Macromolecules* **2012**, *45* (5), 2522-2536. DOI: http://dx.doi.org/10.1021/ma202653k (acccessed 2012/03/02).

(6) Randl, O. G. SQW - A comprehensive user manual. Institut Laue-Langevin (ILL): Grenoble, France, 1996; p ILL internal publication 96RA07T

(7) More exactly, this had to be the fraction of the neutron cross section due to the mobile hydrogen atoms. The problem is here that the coherent cross section is *Q*-dependent, so would be $f_{mob}(Q)$. In the high *Q* limit where coherent and incoherent scattering can be treated as equivalent, one obtains f_{mob} = 0.56. For low *Q* the coherent contribution vanishes and C and O only have negligible incoherent scattering, so f_{mob} = 6/10 = 0.6 is exact. The small variation has no discernible effect on the action of the correction on the NSE data.

(8) Zorn, R.; Arbe, A.; Colmenero, J.; Frick, B.; Richter, D.; Buchenau, U. Neutron scattering study of the picosecond dynamics of polybutadiene and polyisoprene. *Phys. Rev. E: Stat., Nonlinear, Soft Matter Phys.* **1995**, *52*, 781-795. DOI: https://doi.org/10.1103/PhysRevE.52.781.

(9) HSL. *HSL. A collection of Fortran codes for large scale scientific computation.* . 2022. http://www.hsl.rl.ac.uk/ (accessed 2022 10.02.2022).

(10) Pyda, M. *Poly(ethyl methacrylate) (PEMA) Heat Capacity, Enthalpy, Entropy, Gibbs Energy: Datasheet from "The Advanced THermal Analysis System (ATHAS) Databank – Polymer Thermodynamics" Release 2014 in SpringerMaterials* Springer-Verlag Berlin Heidelberg & Marek Pyda, 2014. https://materials.springer.com/polymerthermodynamics/docs/athas_0005 (accessed 2022 14.04.2022).

(11) Tarasov, V. Anisotropic atomic vibrations and the heat capacity of layer and chain structures. In *Dokl. Akad. Nauk. SSSR*, 1955; Vol. 100, p 307.

(12) Tarasov, V. V. Heat capacity of chain and layer structures. *Zh. Fiz. Khim* **1953**, *27*, 1430-1435.

(13) William H. Press; Saul A. Teukolsky; William T. Vetterling; Flannery, B. P. *Numerical Recipes*; Cambridge University Press, 2007.
